# Effects of Angiotensin Receptor-Neprilysin Inhibitors (ARNIs) on the Glucose and Fat Metabolism Biomarkers Leptin and Fructosamine

**DOI:** 10.3390/jcm12093083

**Published:** 2023-04-24

**Authors:** Bernhard Ohnewein, Zornitsa Shomanova, Vera Paar, Albert Topf, Peter Jirak, Lukas Fiedler, Christina Granitz, Vincent Van Almsick, Dilvin Semo, Naufal Zagidullin, Anna-Maria Dieplinger, Juergen Sindermann, Holger Reinecke, Uta C. Hoppe, Rudin Pistulli, Lukas J. Motloch

**Affiliations:** 1 Department for Internal Medicine II, Paracelsus Medical University, 5020 Salzburg, Austria; 2 Department of Cardiology I, Coronary and Peripheral Vascular Disease, Heart Failure, University Hospital Muenster, 48149 Muenster, Germanyrudin.pistulli@ukmuenster.de (R.P.); 3 Department of Internal Medicine, Cardiology, Nephrology and Intensive Care Medicine, Hospital Wiener Neustadt, 2700 Wiener Neustadt, Austria; 4 Department of Internal Diseases, Bashkir State Medical University, Lenin str., 3, 450008 Ufa, Russia; 5 Institute for Nursing Science and Practice, Paracelsus Medical University, 5020 Salzburg, Austria; 6 Medical Faculty, Johannes Kepler University Linz, 4040 Linz, Austria

**Keywords:** leptin, fructosamine, lipid, glucose, metabolism, ARNI, heart failure, HFrEF, neprilysin, valsartan

## Abstract

(1) Background: Heart failure with reduced ejection fraction (HFrEF) remains a major health burden. Angiotensin-Receptor-Neprilysin-Inhibitors (ARNIs) are an established HFrEF therapy which increases natriuretic peptide levels by inhibiting neprilysin. Leptin is a lipid metabolism parameter, which is also involved in glucose metabolism and is suggested to correlate with HF burden. While the hormone also seems to interact with neprilysin, potential associations with ARNI therapy have not been investigated yet. (2) Methods: To study this issue, we measured levels of leptin and fructosamine in consecutive 72 HFrEF patients before initiation of ARNI therapy and 3–6 months after initiation of therapy in two European centers. Biomarker levels were correlated with clinical parameters including ejection fraction, LVEF, and NYHA class. (3) Results: During a follow-up of up to 6 months, clinical parameters improved significantly (LVEF: 30.2 ± 7.8% to 37.6 ± 10.0%, (*p* < 0.001) and a significant improvement of the mean NYHA class with initial 32 patients in NYHA III or IV and 8 patients in NYHA class III/IV during the follow up (*p* < 0.001). The initial NT-proBNP levels of 2251.5 ± 2566.8 pg/mL significantly improved to 1416.7 ± 2145 pg/mL, *p* = 0.008) during follow up. ARNI therapy was also associated with an increase in leptin levels (17.5 ± 23.4 µg/L to 22.9 ± 29.3, *p* < 0.001) and furthermore, affected glucose metabolism indicated by elevation of fructosamine values (333.9 ± 156.8 µmol/L to 454.8 ± 197.8 µmol/L, *p* = 0.013). (4) Conclusion: while in the early phase of therapy, ARNI promotes clinical improvement of HFrEF, and it also seems to affect fat and glucose parameters, indicating significant metabolic implications of this therapy regime.

## 1. Introduction

Heart failure is a major health burden and a leading cause of death with a prevalence of 1.5% to 2% and is estimated to rise to about 3% in the year 2030 [1,2,3]. Shown on an individual level, the lifetime risk is about 20% among adults aged 40 years or older and has a 5-year mortality rate of 60%, which is comparable to many cancers [4,5]. On average, patients have one hospital admission per year after diagnosis and it is the number one reason for the hospital admission of patients aged 65 years or older [6,7]. Interestingly, after a peak in hospital admissions for heart failure in the 1990s, the incidence of first hospital admissions is rising again in the period from 1998 to 2017 [8,9,10].

During the last decade, the development of angiotensin-neprilysin inhibitors (ARNIs) was a milestone in the improvement of heart failure morbidity [11,12]. ARNIs improve the survival rate of heart failure patients by inhibition of neprilysin which leads to an increase in ANP (atrial natriuretic peptide) and BNP (brain natriuretic peptide) levels, which in turn has beneficial effects on preload, inflammation, and fibrosis [13,14]. Neprilysin also affects lipid metabolism by promoting adipogenesis indirectly via BNP inhibition and directly by potentiation of the phosphatidylinositol 3-kinase [15,16,17]. This is of special interest since obesity was described as an independent risk factor for heart failure [18]. A third remarkable metabolic effect of ARNIs was found in glucose metabolism, where the therapy initiation of ARNIs in heart failure patients reduced the need for new insulin therapy by 29% compared to placebo therapy [19]. Increased levels of hbA1c are known to be associated with the progression of heart failure [20]. Here, the mechanisms under discussion are the effects of neprilysin on lipid mobilization from adipose tissue via ANP, indirect effects via adiponectin release, or the beneficial effects of increased BNP levels [19,21,22,23]

The adipokine leptin is secreted predominantly by adipocytes and was initially described as a lipid metabolism marker in its role to reduce food intake and increase energy expenditure [24]. Nevertheless, it seems also to be a promising tool for treating diabetes because it affects glucose metabolism in experimental trials [25]. Leptin improves hepatic insulin sensitivity and glucose uptake in skeletal muscle [26]. Additionally, subcutaneous leptin injection prevents an increase in body fat and improves blood glucose levels in diabetic mice [26,27]. On the other hand, leptin resistance in chronic leptin elevation might blunt the beneficial insulin-sensitizing effects [26,28]. Another parallel to neprilysin is that leptin also has multiple interactions in the cardiovascular system: it is known to be elevated in chronic heart failure [29] and causes its deterioration by activating the sympathetic nervous system, the renin-angiotensin-aldosterone system (RAAS) [30], local and systemic inflammation [30,31] and direct effects on calcium handling [32,33]. On the organ level, chronic elevation causes inflammation, fibrosis, endothelial dysfunction [30,34,35], and left ventricular hypertrophy [30,33] and is associated with increased vascular stiffness [36]. Furthermore, epicardial adipokines directly affect the heart [37]. The interaction of adipose tissue, cardiomyocytes, and smooth muscle cells is reflected by the presence of leptin receptors and the ability of leptin secretion in these tissues [38]. Chronic leptin elevation is associated with adverse cardiovascular outcomes irrespective of BMI levels. Increased levels of leptin were independently associated with a higher incidence of heart failure [39]. In this context, speculations about a possible interaction of leptin and neprilysin in heart failure, glucose and lipid metabolism are considered [30]. Consequently, this could raise further suggestions about possible alterations in the leptin pathway during ARNI therapy in HFrEF. While this also might have possible implications for glucose metabolism, this topic was not evaluated yet.

To examine this issue, we investigated levels of leptin and fructosamine as indicators of measure of non-enzymatic glycation of circulating proteins thus reflecting glucose levels [40] in consecutive 72 HFrEF patients before initiation of ARNI therapy and after 3–6 months of follow-up in 2 European centers. We hypothesized that inhibition of neprilysin would improve HFrEF and affect leptin levels with potential implications for glucose metabolism.

## 2. Materials and Methods

### 2.1. Study Population

In this prospective, observational study patients from two medical centers were investigated. The study included 72 patients with HFrEF of ischemic (*n* = 36) and non-ischemic (*n* = 36) origin. Consecutive patients who provided informed consent and presented at the University Hospital of Salzburg, Austria, or the University Hospital Muenster, Germany with chronic heart failure or progression of chronic heart failure and typical symptoms, elevated N-terminal pro-brain natriuretic peptide (NT-proBNP) > 300 ng/L, left ventricular ejection fraction (LVEF) ≤ 40%, and new treatment with ARNIs were included. Subjects were enrolled between March 2018 and December 2020. The ARNI dose was titrated to 103/97 mg if tolerated. During acute presentation and after three to six months of follow-up, blood samples were taken and clinical investigations including echocardiography were performed. The primary outcomes were the dynamics of NYHA class, LVEF, as well as levels of NT-proBNP, leptin and fructosamine in fasting state under therapy. To assess the effect on the RAAS system, renin and aldosterone levels were measured. No patient was lost during follow-up. Patients were excluded in case of severe valvular heart disease, incompliance, or discontinuation because of severe side effects. Two patients were excluded due to discontinuation of therapy because of side effects.

### 2.2. Blood Sampling

Blood was drawn from a cubical vein and collected in a serum vial to initiate blood coagulation. Notably, 30 min after collection, the blood was centrifuged at 2000× *g* at 4 °C for 20 min. Thereafter, the supernatant was withdrawn and was immediately frozen at −80 °C until further analysis. The parameters glucose, triglycerides, LDL and HDL were obtained as part of our routine blood draw in fasting state. To assess the effect on the RAAS system, we assessed renin and aldosterone levels in the morning after 15 min in seated body posture. In case the test was performed after more than two hours delay, centrifuged, refrigerated blood samples were used.

### 2.3. Biomarker Analysis

Serum concentrations of leptin and fructosamine were measured by a colorimetric detection method. The assays were performed according to the manufacturer’s instructions.

In brief, for leptin enzyme-linked immunosorbent assay (ELISA; RD19100110, BioVendor—Laboratorni medicina a.s., Brno, Czech Republic) the serum samples and the provided standards and quality controls were diluted three-fold with dilution buffer just prior to the assay. Thereafter, 100 µL of the dilutions were applied to the wells. The plates were then incubated for 1 h at room temperature (RT) while they were gently shaken on an orbital shaker. After that, the plates were washed three times with the provided and previously prepared wash solution. Notably, 100 µL of conjugate solution was applied, followed by a 1 h incubation at RT. After the next washing step, 100 µL of substrate solution was added to the wells and was incubated for 10 min at RT without shaking. The color development was stopped by the addition of 100 µL stop solution to the applied substrate. Finally, the optical density (OD) was determined within 5 min with the help of a microplate reader at a wavelength of 450 nm (iMark Microplate Absorbance Reader, Bio-Rad Laboratories, Vienna, Austria).

The fructosamine assay (ab228558; abcam, Cambridge, UK) is based on the reduction of nitro blue tetrazolium (NBT) by fructosamine. For performing the assay, the reagents were prepared according to the manufacturer’s instructions. Thereafter, the fructosamine calibrator and the samples were pipetted into the wells of a clear 96-well plate. The previously prepared reagent mix was added to each sample, followed by the addition of NBT. Then, the plates were incubated for 10 min at 37 °C and the color reaction was gained by adding Fructosamine Buffer B and the incubation at 37 °C for 5 min in the dark. Finally, absorbance was determined by a microplate reader at a wavelength of 530 nm at the time points 5 min (OD_1_) and 15 min (OD_2_). Fructosamine concentrations were calculated using the OD intervals of the calibrator in relation to the samples.

To determine the levels of the lipid parameters LDL, HDL, total cholesterol, and triglycerides, a tube of serum blood was analyzed in the central laboratory of our clinic, where the levels were determined by photometric method (Cobas 8000™; C701™, Fa. Roche Diagnostics, Mannheim, Germany). The same procedure was performed for the fasting glucose levels, which were determined by using an enzymatic reference method with hexokinase. For the measurement of renin and aldosterone, the laboratory test was performed with EDTA blood by a specialized subsection of our laboratory unit, using an ELISA-based photometric method (Sunrise™ absorbance reader, Fa. Tecan Group, Männedorf, Switzerland).

### 2.4. Ethics

The study conformed to the Declaration of Helsinki, had ethics approval by the local ethics committee (protocol codes in Salzburg/Münster: 415-E/2427/7-2019, 2019-011-f-S; dates of approval June 2019; March 2019) and all participants provided written informed consent prior to inclusion.

### 2.5. Statistics

Statistical analyses were performed in IBM SPSS Statistics version 27 (IBM Corp., Armonk, NY, USA). To assess normal distribution of parameters, the Shapiro–Wilk test was used. Normally distributed baseline parameters were presented as mean and standard error of the mean. Means were compared by student’s *t*-test. Not normally distributed baseline parameters were expressed as median and inter-quartile range (IQR) and were compared with Mann–Whitney-U-Test.

Dynamics of concentrations of biomarkers in HFrEF were assessed with dependent *t*-test for normally distributed variables or with Wilcoxon signed rank test for non-normally distributed variables. Categorical variables were expressed as numbers and percentages and compared by using chi-squared test. Correlations were assessed with Pearson’s correlation coefficient or Spearmen’s rank correlation coefficient. All *p*-values were 2-sided and statistical significance was set at below 0.05.

## 3. Results

Baseline characteristics of the 72 included patients (mean age 62 ± 12.7 years, 26.4% women) are shown in Table 1. The mean LVEF was 30.2% (IQR 25.0%;37.0%), of which half had ischemic genesis and half dilatative genesis (*n* = 36/36; 50%/50%). The majority of patients were under sufficient heart failure therapy with angiotensin-converting enzyme inhibitors (ACEI) or angiotensin-1 receptor (ARB) inhibitors (88.9%), betablockers (BB) (87.5%), and mineralocorticoid receptor antagonists (68.1%) before initiation of ARNI therapy. Initially, a high number of patients suffered from severe symptoms (44% NYHA III/IV) and a mean NT-proBNP of 2251 pg/mL (IQR 415-2679). The mean BMI was 28.1 (IQR 23.4;30.9), and 29.2% were obese (defined as a BMI of 30 or higher). The mean LDL was 85.6 mg/dL (IQR 58;97) and the mean triglyceride level was 130.7 mg/dL (IQR 90;148) whereby 44 patients (61.1%) were on statin therapy. Diabetes mellitus was previously diagnosed in 19.4% of patients with a mean HbA1c of 6.9% ± 1.11% in comparison to 5.6% ± 0.39% in non-diabetic patients. On average, patients reached 59% of target ARNI dose of 103/97 mg until follow-up. The median follow-up time was 3.9 (3.1;5.6) months.

### 3.1. Concentrations and Dynamics

**Figure 1:**
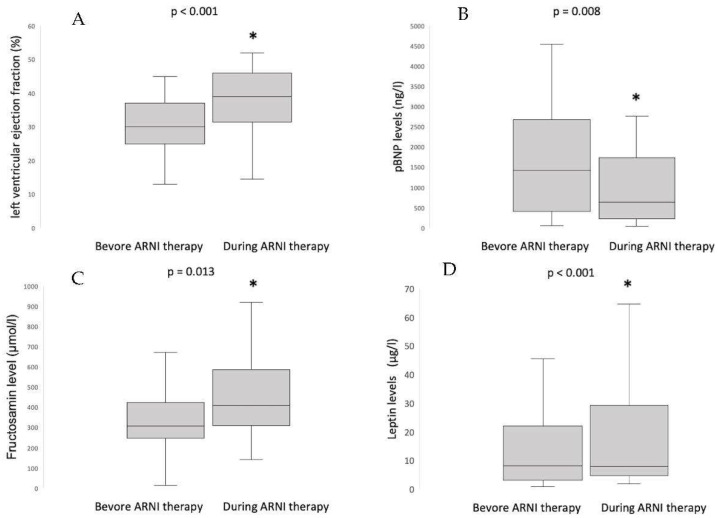
(**A**) Left ventricular ejection fraction, (**B**) plasma concentration of NT-pBNP, (**C**) plasma concentration of fructosamine, and (**D**) plasma concentration of leptin. *: *p* < 0.05.

Biomarker dynamics are shown in Figure 1. Mean baseline levels of leptin were 17.5 ± 23.4 ng/mL, mean baseline levels of fructosamine were 333.9 ± 156.8 µmol/L, and mean baseline levels of NT-proBNP were 2251.5 ± 2566.8 pg/mL. Under HF medical therapy, a significant improvement in ejection fraction from 30.2 ± 7.8% to 37.6 ± 10.0% (*p* ≤ 0.001) and a significant decrease of NT-proBNP to 1416.7 ± 2145 pg/mL (*p* = 0.008) was shown. Interestingly, along with that, a significant increase in leptin levels to 22.9 ± 29.3 (*p* < 0.001) and a significant increase in fructosamine levels to 454.8 ± 197.8 µmol/L (*p* = 0.013) was shown. General lipid and metabolism parameters are shown in Table A1. At follow up, the level of total cholesterol was 150 mg/dL (IQR 132;169; *p* = 0.575), and the LDL cholesterol level was 84 mg/dL (IQR 60;103, *p* = 0.814) and did not significantly change. There was a significant increase in triglyceride levels to 141 mg/dL (IQR 92;178; *p* = 0.009). The hbA1c level at control was 5.7% (IQR 5.4;5.9) and did not significantly differ from the initial level (*p* = 0.521). There was no significant change in mean fasting glucose level during follow-up from 98 mg/dL (IQR 90;115) to 99 mg/dL (IQR 85;114; *p* = 0.391). The baseline level of aldosterone was 81.5 ng/L (IQR 51.5;173.0) and did not change significantly to 129.0 ng/L (IQR 77.1;179.0; *p* = 0.208). The mean level of renin was 28.9 ng/L (IQR 6.5;139.0) and showed a non-significant change to 47.5 ng/L (IQR 6.2; 228.0; *p* = 0.683). There was no significant difference in the aldosterone renin quotient between baseline with 10.1 (IQR 0.5; 11.3) and follow up with 9.2 (IQR 0.4;10.7; *p* = 0.983).

### 3.2. Correlations

**Figure 2:**
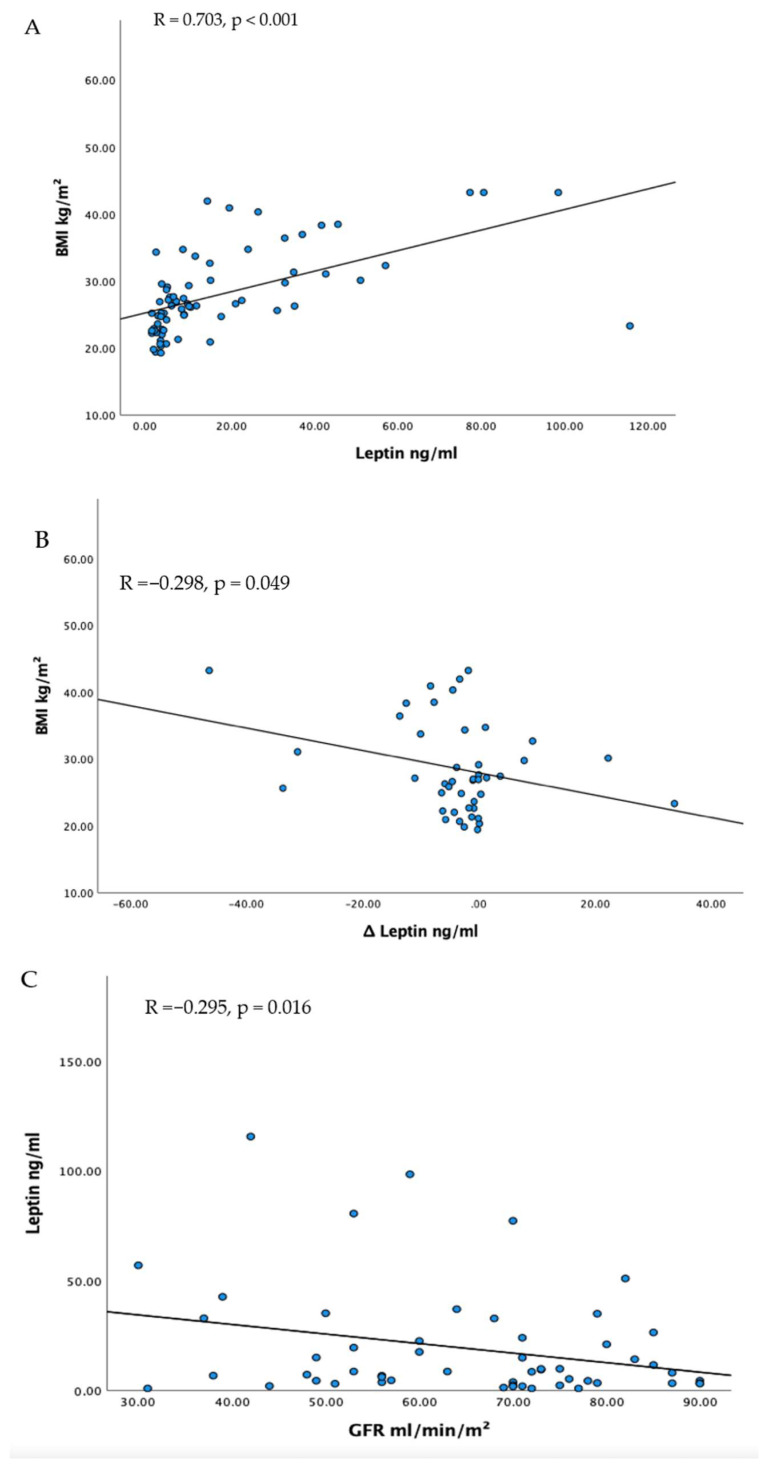
(**A**) Correlation Leptin and BMI (**B**) Correlation Change of Leptin and BMI (**C**) Correlation Leptin and GFR.

Correlations are shown in Figure 2. There was a significant correlation between initial NT-proBNP and initial LVEF (R = 0.416, *p* < 0.001) but there was no significant correlation between the change of NT-proBNP and change of leptin (R = 0.007, *p* = 0.971). Furthermore, we could not find a significant correlation between the change in ejection fraction and the change in leptin (R = 0.183, *p* = 0.235).

We could find a significant correlation between initial leptin levels and initial BMI (R = 0.703, *p* < 0.001). Furthermore, there was a significant correlation between initial BMI and the change in leptin levels during ARNI therapy (R = −0.298, *p* = 0.049). We could show a weak correlation between GFR and initial leptin (R = −0.295, *p* = 0.016) but no correlation with the change in leptin (R = −0.24, *p* = 0.878).

There was no correlation of fructosamine with leptin (R = 0.016, *p* = 0.912) and no correlation between the change of fructosamine with initial leptin (R = 0.029, *p* = 0.902) and the change of leptin (R = 0.145, *p* = 0.606). Interestingly, the change in triglycerides did correlate with the change in fructosamine (R = 0.512, *p* = 0.036). Surprisingly, there was also no correlation of BMI with fructosamine (R = 0.010, *p* = 0.944) and no correlation of fructosamine with GFR (R = 0.117, *p* = 0.414).

## 4. Discussion

Leptin has diverse pathophysiological effects on heart failure, and high levels are associated with increased cardiovascular events. ARNI therapy inhibits neprilysin, which might interact with leptin through multiple pathways. In this study, we investigated the effects of ARNI therapy on leptin and fructosamine levels by analyzing the dynamics after therapy initiation during a short-term follow-up period.

As expected, initiation of ARNI was followed by improvement of left ventricular ejection fraction and levels of NT-proBNP. However, we also observed alterations in the fat metabolism parameter leptin and the glucose metabolism parameter fructosamine.

We found that leptin levels increase after the initiation of ARNI therapy. This result is in accordance with previous studies reporting that leptin levels correlate inversely with the severity of heart failure and that the unloading of the left ventricle increases leptin [41,42]. The impact of leptin elevation on prognosis is unclear. Despite the inverse correlation with NT-proBNP, an increase in leptin is described to be associated with higher mortality, hospitalization rate, or fatal vascular events in coronary artery disease and with the progression of heart failure [43,44].

Deterioration of heart failure by increased levels of aldosterone is caused by increased sodium retention and plasma volume expansion through increased Na+/K+-ATPase activity. This mechanism is of special interest, especially in obesity, where aldosterone is excessively synthesized by adipose tissue. Leptin increases Na+/K+-ATPase activity by stimulating aldosterone secretion, sympathetic nerve activity, and direct activation. The same three mechanisms are known in obesity [30].

Since valsartan develops beneficial effects on aldosterone through the RAAS pathway and neprilysin inhibition might affect aldosterone through its impact on leptin levels, we assessed levels of renin and aldosterone. Interestingly, we could not find a significant change in both parameters. On the one hand, valsartan inhibition was described to reduce aldosterone levels [45], but on the other hand, leptin is known to increase aldosterone levels [46], which finally might lead to neutral results. One might also speculate that the assessment after about four-month of treatments was too early to show significant results.

Since the effects on aldosterone were possibly neutralized by the described mechanisms, there was an improvement in heart failure, which is rather due to the known effects on ANP, BNP, or Substance P. However, our results should be interpreted with caution when dealing with patients during long-term follow-up.

Although the role of leptin on the prognosis of heart failure is unclear, there is rising evidence that the role of leptin in glucose metabolism is important. Diabetes is an independent and strong risk factor for the progression of heart failure [47]. Interestingly, in a post hoc analysis of the PARADIGM-HF trial, an improvement of the diabetes marker HbA1c during long-term follow-up after one and three years was found [19].

Multiple studies have described that leptin increases insulin sensitivity and reduces insulin secretion in animal models. Leptin induces gluconeogenesis and increases glucose uptake and insulin sensitivity in the liver by exerting effects on the central nervous system transmitted by the vagus nerve. More precisely, leptin is able to cross the blood-brain barrier and exert its central effects on leptin receptors of the hypothalamic nucleus arceatus. This results in the regulation of food consumption and increases peripheral energy expenditure and insulin sensitivity. The signaling to the peripheral tissue is transmitted by the central nervous system (e.g., nervus vagus) [26]. This improvement of glucose metabolism might have beneficial effects on glucose levels, represented by fructosamine. Thus, leptin increases glucose metabolism in the skeletal muscles and brown adipose tissue and can improve blood glucose levels in uncontrolled diabetes. Most importantly leptin affects pancreatic cells directly and through the sympathetic nervous system. Thus, pancreatic insulin biosynthesis and secretion are decreased and β-cell proliferation and apoptosis are affected [26,48,49,50,51]. Leptin treatment in diabetic patients with hyperleptinemia improved blood glucose levels clearly [52,53]. We hypothesized that the observed initial increase in leptin after initiation of ARNI therapy might contribute to an improvement of glucose metabolism.

However, in this study, we found an increase in fructosamine, indicating a deterioration of the glucose metabolism marker fructosamine under ARNI therapy. Fructosamine is derived from the carbonylation of a protein with the carbonyl group of glucose. The level of fructosamine increases along with the blood glucose levels and reflects blood glucose levels over the last three weeks. While this seems surprising when considering the literature described above, one should take into account that our study only reflects the initial phase of ARNI therapy (3–6 months of follow-up). This tendency was not reflected in fasting glucose levels at follow up. A possible explanation is a stronger effect of ARNIs on peak glucose levels, which finally resulted in an improvement in fructosamine levels.

Furthermore, we evaluated the dynamics of the lipid metabolism parameters: LDL cholesterol, HDL cholesterol, total cholesterol, and triglycerides. Interestingly, we found a mild but significant increase in triglyceride levels. However, this change did not correlate with the dynamics of leptin. On the other hand, we found a correlation between triglycerides and fructosamine. Thus, one might speculate that this increase could be related to increased insulin resistance, reflected by fructosamine, which is known to contribute to lipolysis and to the deterioration of lipid metabolism [54].

Notably, the early phase of ARNI therapy is characterized by pronounced remodeling processes, which could initially interact with glucose metabolism [55]. Therefore, lower fructosamine levels could be speculated during the long-term follow-up. This might be further supported by a significant but only mild elevation of leptin levels observed during the follow-up in our study. Thus, one might expect leptin levels to rise more significantly during longer follow-up with a consequent counterbalance of initially observed glucose-elevating mechanisms. Nevertheless, while we did not measure leptin and fructosamine during a longer follow-up our speculation should be addressed with caution.

**Figure A1:**
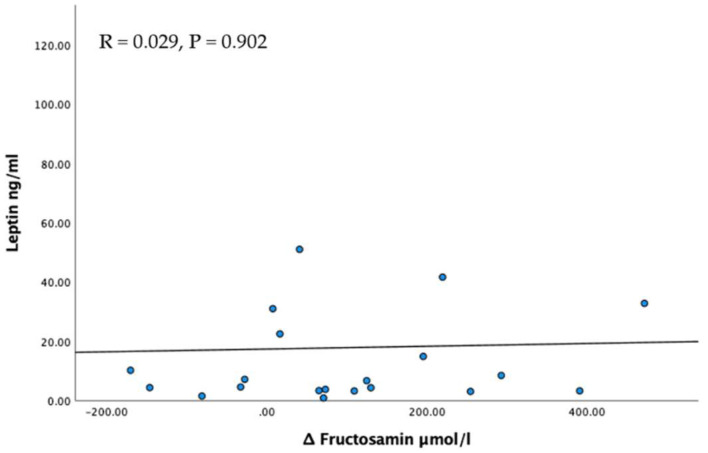
Correlation Leptin with Δ Fructosamine.

Further possible interpretations might be taken into account. The beneficial effects described above are transmitted mainly through the central nervous system and therefore are potentially affected by leptin resistance caused by chronically elevated leptin. Additionally, in chronic heart failure insulin resistance is related to hyperleptinaemia [56,57] This might explain the significant but clinically only irrelevant small improvement of HbA1c during leptin therapy in diabetic patients in a previous study [58]. In our study, we described an increase in leptin during ARNI therapy, but this did not cause an improvement in fructosamine levels. This might be due to leptin resistance, which is reflected by a lack of correlations between initial leptin levels and change in fructosamin as presented in Figure A1. Furthermore, the effect on glucose metabolism was not induced by alterations in the aldosterone renin system.

From a clinical perspective, an improvement in blood glucose levels in heart failure patients should be a main target since the prevalence of diabetes is about 30% in heart failure patients and it is an important risk factor for the progression of heart failure [59,60] As described above, recent studies have shown an improvement in blood glucose levels in the long term. ARNIs increase insulin sensitivity in hypertensive patients [61] and increased glucose uptake was shown in an animal model [62]. Taking this into account, the beneficial effects of ARNI therapy on glucose metabolism might outweigh it in the long term, yet our study might emphasize the tight control of blood glucose levels in patients with diabetes mellitus in the initial phase of ARNI therapy.

In conclusion, we were able to investigate various possible pathways of interactions of neprilysin inhibition and glucose and lipid metabolism. Thus, we intended to generate a better understanding of the metabolic effects of ARNIs, which might contribute to an improved prediction of their impact on prognostic important glucose and lipid parameters in clinical settings.

## 5. Limitations

Although we evaluated the known parameters that affect leptin, it is important to consider further unknown parameters which might affect leptin and fructosamine dynamics. Due to the lack of a control arm, a causal interpretation of the results is difficult since the natural course of the disease might affect the biomarkers to some extent. However, in previous trials, the dynamics of leptin in heart failure without ARNIs were well described [29,63]. Although we had no loss of follow-up, two patients had to stop ARNI therapy due to side effects and therefore were excluded. It is unlikely that these two patients would have affected the results in a relevant way since our results show robust significance.

## 6. Conclusions

In summary, we found surprising dynamics in fat and glucose metabolism during the early phase after ARNI therapy initiation, indicating an increase in leptin during the improvement of HFrEF. The increase in fructosamine in the early phase of ARNI therapy might reflect an initial worsening of glucose metabolism in the early phase of ARNI therapy. However, this observation seemed not to correlate with affected leptin levels.

## Figures and Tables

**Table 1:** Baseline characteristics; median, lower and upper quartiles or standard deviation (Q1; Q3 or SD) and *n* (%).

Baseline Characteristics	Specification	Results	Q1; Q3/SD or %
Demographics	Age (years)	62.0	(±12.7)
	Gender (male/female)	54/20	(73;27)
Medical history	Ischemic Cardiomyopathy	36	(50.0)
	Non-ischemic Cardiomyopathy	36	(50.0)
	Atrial fibrillation *	20	(26.8)
	Dyslipidemia	42	(56.3)
	Diabetes Mellitus	24	(32.4)
	Hypertension	39	(54.9)
	Chronic kidney disease **	22	(30.6)
	History of smoking	36	(51.4)
Clinical Measurement	BMI	28.1	(23.4;30.9)
	SBP (mmHg)	125	(113,137)
	Heart rate (bpm)	72	(60;81)
	LVEF (%)	30.2	(25.0;37.0)
	LVEDD (mm)	60.4	(±8.8)
Treatment	ACE/ARB (before ARNI)	64	(88.9)
	BB	63	(87.5)
	MRA	49	(68.1)
	Statin	44	(61.1)
	Ezetimib	7	(9.3)
	Loop Diuretics	40	(55.6)
	Thiazides	3	(4.2)
	Metformin	20	(27.7)
	GLP-1 Inhibitors	7	(9.2)
	Insulin	5	(6.7)
Laboratory	NT-proBNP (ng/L)	2251	(415;2679)
	Hemoglobin g/L	13.9	(±1.8)
	eGFR (mL/min/1.73 m^2^)	69.7	(56.0;87.0)
	Total cholesterol (mg/dL)	156.3	(122;176)
	Triglyceride (mg/dL)	130.7	(90;148)
	LDL (mg/dL)	85.6	(58;97)
	HDL (mg/dL)	51.5	(37;58)
	HbA1c (%)	5.9	(5.4;6.1)
	CRP (mg/dL)	0.7	(0.38;0.63)

* Including the history of paroxysmal, persistent or permanent atrial fibrillation; ** eGFR < 60 mL/min/1.73 m^2^.

## Data Availability

The data presented in this study are available on request from the corresponding author.

## Appendix A

**Table A1:** Metabolism parameters at baseline and follow up. *: *p* < 0.05.

Metabolism Parameter	Level Baseline Median	Level Follow Up Median	*p*-Value
LDL (mg/dL)	85 (58;97)	84 (60;103)	0.814
HDL (mg/dL)	51 (37;58)	49 (40;60)	0.413
Total cholesterol (mg/dL)	156 (122;176)	150 (132;169)	0.575
Triglycerides (mg/dL)	130 (90;148)	141 (92;178)	0.009 *
Blood glucose (mg/dL)	98 (90;115)	99 (85;114)	0.391
hbA1c (%)	5.9 (5.4;6.1)	5.7 (5.4;5.9)	0.521
Renin (ng/L)	28.9 (6.5;139.0)	47.5 (6.2; 228.0)	0.683
Aldosterone (ng/L)	81.5 (51.5;173.0)	129.0 (77.1;179.0)	0.208

## References

[1] Virani S.S., Alonso A., Benjamin E.J., Bittencourt M.S., Callaway C.W., Carson A.P., Chamberlain A.M., Chang A.R., Cheng S., Delling F.N. (2020). Heart Disease and Stroke Statistics-2020 Update: A Report From the American Heart Association. Circulation.

[2] Conrad N., Judge A., Tran J., Mohseni H., Hedgecott D., Crespillo A.P., Allison M., Hemingway H., Cleland J.G., McMurray J.J.V. (2018). Temporal trends and patterns in heart failure incidence: A population-based study of 4 million individuals. Lancet.

[3] Heidenreich P.A., Albert N.M., Allen L.A., Bluemke D.A., Butler J., Fonarow G.C., Ikonomidis J.S., Khavjou O., Konstam M.A., Maddox T.M. (2013). Forecasting the impact of heart failure in the United States: A policy statement from the American Heart Association. Circ. Heart Fail.

[4] Lloyd-Jones D.M., Larson M.G., Leip E.P., Beiser A., D’Agostino R.B., Kannel W.B., Murabito J.M., Vasan R.S., Benjamin E.J., Levy D. (2002). Lifetime risk for developing congestive heart failure: The Framingham Heart Study. Circulation.

[5] Tsao C.W., Lyass A., Enserro D., Larson M.G., Ho J.E., Kizer J.R., Gottdiener J.S., Psaty B.M., Vasan R.S. (2018). Temporal Trends in the Incidence of and Mortality Associated With Heart Failure With Preserved and Reduced Ejection Fraction. JACC Heart Fail.

[6] Barasa A., Schaufelberger M., Lappas G., Swedberg K., Dellborg M., Rosengren A. (2014). Heart failure in young adults: 20-year trends in hospitalization, aetiology, and case fatality in Sweden. Eur. Heart J..

[7] Hall M.J., DeFrances C.J., Williams S.N., Golosinskiy A., Schwartzman A. (2010). National Hospital Discharge Survey: 2007 summary. Natl. Health Stat. Rep..

[8] Jhund P.S., Macintyre K., Simpson C.R., Lewsey J.D., Stewart S., Redpath A., Chalmers J.W., Capewell S., McMurray J.J. (2009). Long-term trends in first hospitalization for heart failure and subsequent survival between 1986 and 2003: A population study of 5.1 million people. Circulation.

[9] Lawson C.A., Zaccardi F., Squire I., Ling S., Davies M.J., Lam C.S.P., Mamas M.A., Khunti K., Kadam U.T. (2019). 20-year trends in cause-specific heart failure outcomes by sex, socioeconomic status, and place of diagnosis: A population-based study. Lancet Public Health.

[10] McDonagh T.A., Metra M., Adamo M., Gardner R.S., Baumbach A., Böhm M., Burri H., Butler J., Čelutkienė J., Chioncel O. (2021). 2021 ESC Guidelines for the diagnosis and treatment of acute and chronic heart failure. Eur. Heart J..

[11] McMurray J.J.V., Packer M., Desai A.S., Gong J., Lefkowitz M.P., Rizkala A.R., Rouleau J.L., Shi V.C., Solomon S.D., Swedberg K. (2014). Angiotensin–Neprilysin Inhibition versus Enalapril in Heart Failure. N. Engl. J. Med..

[12] McMurray J.J.V., Solomon S.D., Inzucchi S.E., Køber L., Kosiborod M.N., Martinez F.A., Ponikowski P., Sabatine M.S., Anand I.S., Bělohlávek J. (2019). Dapagliflozin in Patients with Heart Failure and Reduced Ejection Fraction. N. Engl. J. Med..

[13] von Lueder T.G., Wang B.H., Kompa A.R., Huang L., Webb R., Jordaan P., Atar D., Krum H. (2015). Angiotensin receptor neprilysin inhibitor LCZ696 attenuates cardiac remodeling and dysfunction after myocardial infarction by reducing cardiac fibrosis and hypertrophy. Circ. Heart Fail.

[14] Pu Q., Amiri F., Gannon P., Schiffrin E.L. (2005). Dual angiotensin-converting enzyme/neutral endopeptidase inhibition on cardiac and renal fibrosis and inflammation in DOCA-salt hypertensive rats. J. Hypertens.

[15] Kim J., Han D., Byun S.H., Kwon M., Cho S.J., Koh Y.H., Yoon K. (2017). Neprilysin facilitates adipogenesis through potentiation of the phosphatidylinositol 3-kinase (PI3K) signaling pathway. Mol. Cell. Biochem..

[16] Sengenes C., Stich V., Berlan M., Hejnova J., Lafontan M., Pariskova Z., Galitzky J. (2002). Increased lipolysis in adipose tissue and lipid mobilization to natriuretic peptides during low-calorie diet in obese women. Int. J. Obes. Relat. Metab. Disord..

[17] Bordicchia M., Liu D., Amri E.Z., Ailhaud G., Dessì-Fulgheri P., Zhang C., Takahashi N., Sarzani R., Collins S. (2012). Cardiac natriuretic peptides act via p38 MAPK to induce the brown fat thermogenic program in mouse and human adipocytes. J. Clin. Investig..

[18] Ndumele C.E., Matsushita K., Lazo M., Bello N., Blumenthal R.S., Gerstenblith G., Nambi V., Ballantyne C.M., Solomon S.D., Selvin E. (2016). Obesity and Subtypes of Incident Cardiovascular Disease. J. Am. Heart Assoc..

[19] Seferovic J.P., Claggett B., Seidelmann S.B., Seely E.W., Packer M., Zile M.R., Rouleau J.L., Swedberg K., Lefkowitz M., Shi V.C. (2017). Effect of sacubitril/valsartan versus enalapril on glycaemic control in patients with heart failure and diabetes: A post-hoc analysis from the PARADIGM-HF trial. Lancet Diabetes Endocrinol..

[20] Echouffo-Tcheugui J.B., Ndumele C.E., Zhang S., Florido R., Matsushita K., Coresh J., Skali H., Shah A.M., Selvin E. (2022). Diabetes and Progression of Heart Failure: The Atherosclerosis Risk In Communities (ARIC) Study. J. Am. Coll. Cardiol..

[21] Birkenfeld A.L., Boschmann M., Moro C., Adams F., Heusser K., Franke G., Berlan M., Luft F.C., Lafontan M., Jordan J. (2005). Lipid mobilization with physiological atrial natriuretic peptide concentrations in humans. J. Clin. Endocrinol. Metab..

[22] Coué M., Badin P.M., Vila I.K., Laurens C., Louche K., Marquès M.A., Bourlier V., Mouisel E., Tavernier G., Rustan A.C. (2015). Defective Natriuretic Peptide Receptor Signaling in Skeletal Muscle Links Obesity to Type 2 Diabetes. Diabetes.

[23] Birkenfeld A.L., Boschmann M., Engeli S., Moro C., Arafat A.M., Luft F.C., Jordan J. (2012). Atrial natriuretic peptide and adiponectin interactions in man. PLoS ONE.

[24] Obradovic M., Sudar-Milovanovic E., Soskic S., Essack M., Arya S., Stewart A.J., Gojobori T., Isenovic E.R. (2021). Leptin and Obesity: Role and Clinical Implication. Front. Endocrinol..

[25] Seferovic J.P., Solomon S.D., Seely E.W. (2020). Potential mechanisms of beneficial effect of sacubitril/valsartan on glycemic control. Ther. Adv. Endocrinol. Metab..

[26] Amitani M., Asakawa A., Amitani H., Inui A. (2013). The role of leptin in the control of insulin-glucose axis. Front. Neurosci..

[27] Wang Y., Asakawa A., Inui A., Kosai K. (2010). Leptin gene therapy in the fight against diabetes. Expert. Opin. Biol. Ther..

[28] Belke D.D., Larsen T.S., Gibbs E.M., Severson D.L. (2000). Altered metabolism causes cardiac dysfunction in perfused hearts from diabetic (db/db) mice. Am. J. Physiol. Endocrinol. Metab..

[29] Schulze P.C., Kratzsch J., Linke A., Schoene N., Adams V., Gielen S., Erbs S., Moebius-Winkler S., Schuler G. (2003). Elevated serum levels of leptin and soluble leptin receptor in patients with advanced chronic heart failure. Eur. J. Heart Fail.

[30] Packer M. (2018). Leptin-Aldosterone-Neprilysin Axis: Identification of Its Distinctive Role in the Pathogenesis of the Three Phenotypes of Heart Failure in People With Obesity. Circulation.

[31] Pérez-Pérez A., Sánchez-Jiménez F., Vilariño-García T., Sánchez-Margalet V. (2020). Role of Leptin in Inflammation and Vice Versa. Int. J. Mol. Sci..

[32] Na T., Dai D.Z., Tang X.Y., Dai Y. (2007). Upregulation of leptin pathway correlates with abnormal expression of SERCA2a, phospholamban and the endothelin pathway in heart failure and reversal by CPU86017. Naunyn Schmiedebergs Arch. Pharmacol..

[33] Poetsch M.S., Strano A., Guan K. (2020). Role of Leptin in Cardiovascular Diseases. Front. Endocrinol..

[34] Martínez-Martínez E., Miana M., Jurado-López R., Bartolomé M.V., Souza Neto F.V., Salaices M., López-Andrés N., Cachofeiro V. (2014). The potential role of leptin in the vascular remodeling associated with obesity. Int. J. Obes..

[35] Martínez-Martínez E., Jurado-López R., Valero-Muñoz M., Bartolomé M.V., Ballesteros S., Luaces M., Briones A.M., López-Andrés N., Miana M., Cachofeiro V. (2014). Leptin induces cardiac fibrosis through galectin-3, mTOR and oxidative stress: Potential role in obesity. J. Hypertens..

[36] Singhal A., Farooqi I.S., Cole T.J., O’Rahilly S., Fewtrell M., Kattenhorn M., Lucas A., Deanfield J. (2002). Influence of leptin on arterial distensibility: A novel link between obesity and cardiovascular disease?. Circulation.

[37] Patel V.B., Mori J., McLean B.A., Basu R., Das S.K., Ramprasath T., Parajuli N., Penninger J.M., Grant M.B., Lopaschuk G.D. (2016). ACE2 Deficiency Worsens Epicardial Adipose Tissue Inflammation and Cardiac Dysfunction in Response to Diet-Induced Obesity. Diabetes.

[38] Ghantous C.M., Azrak Z., Hanache S., Abou-Kheir W., Zeidan A. (2015). Differential Role of Leptin and Adiponectin in Cardiovascular System. Int. J. Endocrinol..

[39] Wannamethee S.G., Shaper A.G., Whincup P.H., Lennon L., Sattar N. (2011). Obesity and risk of incident heart failure in older men with and without pre-existing coronary heart disease: Does leptin have a role?. J. Am. Coll. Cardiol..

[40] Gounden V., Ngu M., Anastasopoulou C., Jialal I. (2022). Fructosamine. StatPearls.

[41] Faxén U.L., Hage C., Andreasson A., Donal E., Daubert J.C., Linde C., Brismar K., Lund L.H. (2017). HFpEF and HFrEF exhibit different phenotypes as assessed by leptin and adiponectin. Int. J. Cardiol..

[42] McGaffin K.R., Moravec C.S., McTiernan C.F. (2009). Leptin signaling in the failing and mechanically unloaded human heart. Circ. Heart Fail.

[43] Mayer O., Bruthans J., Seidlerová J., Gelžinský J., Kučera R., Karnosová P., Mateřánková M., Wohlfahrt P., Cífková R., Filipovský J. (2022). high leptin status indicates an increased risk of mortality and heart failure in stable coronary artery disease. Nutr. Metab. Cardiovasc. Dis..

[44] Bobbert P., Jenke A., Bobbert T., Kühl U., Rauch U., Lassner D., Scheibenbogen C., Poller W., Schultheiss H.P., Skurk C. (2012). High leptin and resistin expression in chronic heart failure: Adverse outcome in patients with dilated and inflammatory cardiomyopathy. Eur. J. Heart Fail.

[45] Giles T.D., Bakris G., Oparil S., Weber M.A., Li H., Mallick M., Bharucha D.B., Chen C., Ferguson W.G., Sorin J. (2015). Correlations of plasma renin activity and aldosterone concentration with ambulatory blood pressure responses to nebivolol and valsartan, alone and in combination, in hypertension. J. Am. Soc. Hypertens.

[46] Huby A.C., Antonova G., Groenendyk J., Gomez-Sanchez C.E., Bollag W.B., Filosa J.A., Belin de Chantemèle E.J. (2015). Adipocyte-Derived Hormone Leptin Is a Direct Regulator of Aldosterone Secretion, Which Promotes Endothelial Dysfunction and Cardiac Fibrosis. Circulation.

[47] Pocock S.J., Wang D., Pfeffer M.A., Yusuf S., McMurray J.J., Swedberg K.B., Ostergren J., Michelson E.L., Pieper K.S., Granger C.B. (2006). Predictors of mortality and morbidity in patients with chronic heart failure. Eur. Heart J..

[48] Mizuno A., Murakami T., Otani S., Kuwajima M., Shima K. (1998). Leptin affects pancreatic endocrine functions through the sympathetic nervous system. Endocrinology.

[49] Seufert J. (2004). Leptin effects on pancreatic beta-cell gene expression and function. Diabetes.

[50] Seufert J., Kieffer T.J., Habener J.F. (1999). Leptin inhibits insulin gene transcription and reverses hyperinsulinemia in leptin-deficient ob/ob mice. Proc. Natl. Acad. Sci. USA.

[51] Marroquí L., Gonzalez A., Ñeco P., Caballero-Garrido E., Vieira E., Ripoll C., Nadal A., Quesada I. (2012). Role of leptin in the pancreatic β-cell: Effects and signaling pathways. J. Mol. Endocrinol..

[52] Park J.Y., Chong A.Y., Cochran E.K., Kleiner D.E., Haller M.J., Schatz D.A., Gorden P. (2008). Type 1 diabetes associated with acquired generalized lipodystrophy and insulin resistance: The effect of long-term leptin therapy. J. Clin. Endocrinol. Metab..

[53] Ebihara K., Masuzaki H., Nakao K. (2004). Long-term leptin-replacement therapy for lipoatrophic diabetes. N. Engl. J. Med..

[54] Zhao J., Wu Y., Rong X., Zheng C., Guo J. (2020). Anti-Lipolysis Induced by Insulin in Diverse Pathophysiologic Conditions of Adipose Tissue. Diabetes Metab. Syndr. Obes..

[55] Yu F., McLean B., Badiwala M., Billia F. (2022). Heart Failure and Drug Therapies: A Metabolic Review. Int. J. Mol. Sci..

[56] Doehner W., Rauchhaus M., Godsland I.F., Egerer K., Niebauer J., Sharma R., Cicoira M., Florea V.G., Coats A.J., Anker S.D. (2002). Insulin resistance in moderate chronic heart failure is related to hyperleptinaemia, but not to norepinephrine or TNF-alpha. Int. J. Cardiol..

[57] Riehle C., Abel E.D. (2016). Insulin Signaling and Heart Failure. Circ. Res..

[58] Moon H.S., Matarese G., Brennan A.M., Chamberland J.P., Liu X., Fiorenza C.G., Mylvaganam G.H., Abanni L., Carbone F., Williams C.J. (2011). Efficacy of metreleptin in obese patients with type 2 diabetes: Cellular and molecular pathways underlying leptin tolerance. Diabetes.

[59] Kenny H.C., Abel E.D. (2019). Heart Failure in Type 2 Diabetes Mellitus. Circ. Res..

[60] Maggioni A.P., Dahlström U., Filippatos G., Chioncel O., Leiro M.C., Drozdz J., Fruhwald F., Gullestad L., Logeart D., Metra M. (2010). EURObservational Research Programme: The Heart Failure Pilot Survey (ESC-HF Pilot). Eur. J. Heart Fail.

[61] Jordan J., Stinkens R., Jax T., Engeli S., Blaak E.E., May M., Havekes B., Schindler C., Albrecht D., Pal P. (2017). Improved Insulin Sensitivity With Angiotensin Receptor Neprilysin Inhibition in Individuals with Obesity and Hypertension. Clin. Pharmacol. Ther..

[62] Wang M.Y., Chen L., Clark G.O., Lee Y., Stevens R.D., Ilkayeva O.R., Wenner B.R., Bain J.R., Charron M.J., Newgard C.B. (2010). Leptin therapy in insulin-deficient type I diabetes. Proc. Natl. Acad. Sci. USA.

[63] Doehner W., Pflaum C.D., Rauchhaus M., Godsland I.F., Egerer K., Cicoira M., Florea V.G., Sharma R., Bolger A.P., Coats A.J. (2001). Leptin, insulin sensitivity and growth hormone binding protein in chronic heart failure with and without cardiac cachexia. Eur. J. Endocrinol..

